# A real-time prediction interval correction method with an unscented Kalman filter for settlement monitoring of a power station dam

**DOI:** 10.1038/s41598-023-31182-x

**Published:** 2023-03-11

**Authors:** Ting Zhou, Yuxin Jie, Yingjie Wei, Yanyi Zhang, Hui Chen

**Affiliations:** 1 State Key Laboratory of Hydroscience and Engineering, Tsinghua University, Beijing, 100084 People’s Republic of China; 2 School of Engineering and Technology, China University of Geosciences (Beijing), Beijing, 100083 People’s Republic of China; 3 China Institute of Water Resources and Hydropower Research, Beijing, 100038 People’s Republic of China

**Keywords:** Engineering, Mathematics and computing

## Abstract

A prediction interval (PI) method is developed to quantify the model uncertainty of embankment settlement prediction. Traditional PIs are constructed based on specific past period information and remain unchanged; hence, they neglect discrepancies between previous calculations and new monitoring data. In this paper, a real-time prediction interval correction method is proposed. Time-varying PIs are built by continuously incorporating new measurements into model uncertainty calculations. The method consists of trend identification, PI construction, and real-time correction. Primarily, trend identification is carried out by wavelet analysis to eliminate early unstable noise and determine the settlement trend. Then, the Delta method is applied to construct PIs based on the characterized trend, and a comprehensive evaluation index is introduced. The model output and the upper and lower bounds of the PIs are updated by the unscented Kalman filter (UKF). The effect of the UKF is compared with that of the Kalman filter (KF) and extended Kalman filter (EKF). The method was demonstrated in the Qingyuan power station dam. The results show that the time-varying PIs based on trend data are smoother than those based on original data with better evaluation index scores. Also, the PIs are not affected by local anomalies. The proposed PIs are consistent with the actual measurements, and the UKF performs better than the KF and EKF. The approach has the potential to provide more reliable embankment safety assessments.

## Introduction

Embankment settlement prediction is crucial to engineering design, construction, and usage, especially for high-fill engineering^1^. However, embankment settlement is a nonlinear, complicated problem and can be affected by multiple factors. The nonlinearity of the embankment settlement system arises from the viscous, plastic, and rheological behaviors of construction materials. The constitutive relationship can be expressed by complex forms of power, exponential, and fractional derivative functions. In addition, large amounts of monitoring data can be obtained due to multiple and high-rate sensor development. Incorporating comprehensively mined sensor data into a reasonable physical-guided prediction model is worth investigating. The traditional tool of settlement prediction for structures is point forecasting. This is a deterministic method with simple and intuitive features. However, it does not include uncertainty information due to various factors that may influence the estimation. Such neglect can lead to disagreement between prediction and reality in complex engineering problems. Additionally, the evaluation index of point prediction may not be adequate to make safety decisions.

Prediction intervals (PIs) are developed to quantify the level of uncertainty during estimation and can be used to overcome the limitation of point forecasts. PIs can provide more information with an accuracy indication or a confidence level (1−∝)%. Methods for constructing PIs mainly include statistical models, fuzzy theory models, machine learning models, artificial intelligence models, and combined methods. Generally, Delta, Bayesian, bootstrap, and mean–variance estimation are four commonly used statistical models. The Delta method assumes the probability distribution of the data errors. The method contains Jacobian calculations and linearization by the Taylor expansion. The Delta method has a wide range of applications in parameterized models^2,3^. The Bayesian technique is developed to construct PIs with a Bayesian framework. The method requires a computationally complex Hessian matrix calculation^4^. The mean-covariance method requires large datasets for the entire probability distribution estimation^5^. Bootstrap is a resampling method and requires data assumptions. The calculation is simple and accurate but may be time-consuming when there is a large amount of data^6,7^. Fuzzy theory extracts features of fuzzy information in uncertain systems and then makes a prediction. It includes the gray model^8^, fuzzy reasoning^9,10^, etc. Machine learning (ML) models, such as support vector machines (SVMs), neural networks (NNs), and extreme learning machines (ELMs), are usually used for forecasting^11–13^. Neural network (NN) methods are powerful for generating PIs due to their learning abilities^14–16^. Standard NNs are based on one-order gradient estimation, and the performance of PIs may be influenced by reasonable NN model choice and development^17,18^.

There are also many studies that focus on hybrid intelligent methods. For example, models that combine statistical methods, such as Delta and Bayesian methods, and neural networks (NNs) have been developed in recent years^19^. In addition, optimization algorithms are incorporated into traditional prediction methods for better model configuration, e.g., particle swarm optimization can be embedded in NNs for network modification^20^. The newly proposed lower upper bound estimation method uses the simulated annealing algorithm to optimize the bound of the prediction interval in NNs^21^. A PSO-optimized SVM was proposed to estimate the PIs in the field of electricity^22^. There are currently not many applications of PIs in the field of embankment settlement. Interval risk analysis has been carried out for gravity dam stability estimation^23^. A recent study introduced PI construction for concrete dams utilizing the combined method of bootstrap, a least-square support vector machine, and a neural network^24^. Among the studies, most of the intelligent PI methods are data-driven. The settlement of an embankment is more likely a physically driven problem influenced by constitutive relationships. In addition, the abovementioned PIs are established based on historical datasets, and new measurements are not involved in PI construction^20–24^. Therefore, updated PIs that consider real-time measurements can be developed to improve prediction accuracy.

The Kalman filter (KF) method has been widely applied for real-time updating. The KF was first proposed in 1960^25^, and it can be regarded as a recursive form of Gaussian least square estimation^26^. The method incorporates the error between the measurement and prediction in each time step for real-time prediction correction. The KF has been widely used in areas of sensor fusing, robotics, navigation, etc. In embankment settlement, the applications of the KF include spatiotemporal prediction^27^, anomaly monitoring data detection^28^, and model parameter modification^29^. Generally, the KF method is suitable for linear systems. Therefore, the extended Kalman filter (EKF) was developed and is a widely used technique to cope with nonlinear problems in the real world. The Taylor expansion has been applied to the EKF for linearization; however, the Jacobian matrix may be difficult to calculate or may not exist in some systems^30^. Some improved EKF methods have been developed for nonlinear applications, such as using set membership^31^, adaptive techniques^32^, and enhanced error schemes^33^. The recently developed unscented Kalman filter (UKF) has become a promising method to extend the application of the EKF. It generates sigma points to approximate the distribution via the unscented transform technique^34^. The UKF method is derivative-free and is adaptive to systems with high nonlinearities and discontinuities^35–39^. Some researchers have modified the improved UKF algorithm by using random weighting^40–42^, INS/GNSS integration^43,44^, and adaptive strategies^42^. An iterated square root UKF was proposed for a nonlinear spring-mass-dashpot system^45^. The model parameter of a reinforced concrete structure was updated by a constrained UKF with experimental data^46^. The finite element model parameters of pine flat concrete were updated by the UKF^47,48^. Due to the development of computing power, the cubature Kalman filter (CKF) was proposed and developed to solve highly nonlinear problems. The CKF and the improved versions show satisfactory performance in navigation systems^18,49–52^. The above studies are mainly focused on updating point prediction or estimation. The EKF or UKF can be involved in PIs for uncertainty qualification. For instance, Guan proposed hybrid EKF and UKF-trained NNs to estimate errors for load PIs^53^. The model is mainly a data-driven technique and is more suitable for energy systems^54^. Zhang applied an improved EKF for price interval estimation by combining modified U-D factorization with a decoupled EKF^55^. The method can improve the EKF performance to some degree, while Jacobian calculations are still inevitable. Generally, research on updating PIs by the UKF is not extensive.

Among the abovementioned studies, most of the conventional PIs are constructed based on historical databases and are not calibrated by new monitoring data. In this case, the previous PIs may be reconsidered to cover new information and meet real-time prediction targets, which is computationally expensive and time-consuming. Therefore, it is promising to build time-varying PIs to perform continuous estimation and decrease the prediction uncertainty without reconstructing models. In comparison with data-driven intelligence model, the UKF-updated PIs based on the constitutive relationship may have certain advantages due to the physically driven and nonlinear characteristics of embankment settlement.

In this paper, we propose a real-time interval prediction correction method with the UKF for embankment settlement estimation. The method focuses on the updating of PIs based on a nonlinear viscoelastic physical model. The proposed PIs are time-varying and can iteratively incorporate new monitoring data into the previously established model. The method combines uncertainty estimation with real-time monitoring correction to improve prediction performance. Consequently, it is promising to reduce the deviation between the state estimation and the actual measurement.

The implementation of the approach is as follows. (1) The discrete wavelet transform is applied to extract the settlement trend based on actual measurements. The major trend of the settlement is identified to eliminate noise and unstable settlement information. In addition, clustering-change point analysis is carried out to remove early unstable settlement information. (2) Then, the Delta method is applied to estimate prediction intervals (PIs) based on a parametric regression model. An assessment index for PIs is introduced afterward. (3) The goal of the study is to update and adjust the model based on uncertainty instead of the deterministic magnitude in previous investigations. Finally, the method is employed for real pumped-storage power station settlement prediction engineering.

## Methodology

### Wavelet analysis

The Fourier series is mainly aimed at stationary signals but may behave poorly when dealing with signals with local characteristics. Wavelet analysis is developed on the basis of the Fourier transform. Wavelets are bases that can be translated, stretched, and compressed. The scale function $\phi$ and wavelet function $\varphi$ are used to generate a family of functions for decomposing and reconstructing signals. The main steps of signal $y = f(t)$ processing can be described as follows^56^:

Step 1: *Sampling*.

Set the decomposition level $j$ as the maximum $J$ value, where $0 \le k \le 2^{J} - 1$. Then, the approximation of $f(t)$ is calculated, where *a* is the discomposing coefficient at different scale levels.

1 $$f_{J} (x) = \sum\limits_{k \in Z} {a_{k}^{J} \phi (} 2^{J} x - k)$$

Step 2: *Decomposition*.$f_{J}$ is discomposed as Eq. (2). Where $w_{j - 1} {, }...{, }w_{J - 1}$ are components of signals $f_{J}$ under different decomposition levels.

2 $$f_{J} = w_{J - 1} + ... + w_{j - 1} + f_{j - 1}$$

The specific expressions of $w_{j - 1}$ and $f_{j - 1}$ are as follows:

3 $$w_{j - 1} = \sum\limits_{l \in Z} {b_{l}^{j - 1} \psi (2^{j - 1} x - l)}$$

4 $$f_{j - 1} = \sum\limits_{l \in Z} {a_{l}^{j - 1} \phi (2^{j - 1} x - l)}$$

Step 3: *Processing.*

The coefficients $b_{k}^{j}$ are modified based on the decomposed signal. The coefficients that exceed the threshold are set to 0 to filter unnecessary components.

5 $$f_{J} = \sum\limits_{j = 0}^{J - 1} {w_{j - 1} } + f_{0} = \sum\limits_{j = 0}^{J - 1} {(\sum\limits_{k \in Z} {b_{k}^{j} \psi (2^{j} x - k)} } ) + \sum\limits_{l \in Z} {a_{k}^{0} \phi (x - k)}$$

Step 4: *Reconstruction*.

The reconstructed signals are as follows.

6 $$f_{J} = \sum\limits_{k \in Z} {a_{k}^{J} \phi (2^{j - 1} x - k)}$$

**Figure 1:**
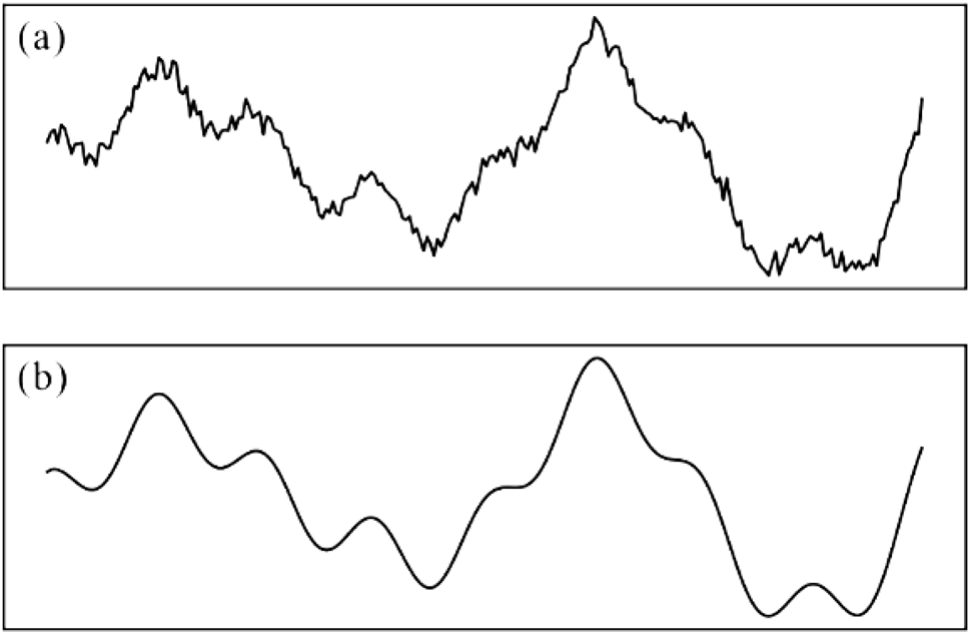
Denoising effect of wavelet analysis: (**a**) original signal; and (**b**) signal processed with wavelet analysis.

Wavelet analysis can be applied to information compression and trend identification. Figure 1 illustrates the denoising effect of wavelet analysis on nonstationary signals. The processed signals are smoother and can reveal the general tendency of information without high frequency fluctuations.

### PI construction

We use the Delta method to construct PIs in this study^57,58^; i.e., consider a parametric model $f(x_{i} ;\;\theta *)$, where $x_{i}$ represents the sample and $\theta *$ is the model parameter. The dimensions of the samples and parameters are $n$ and $p$, respectively. The actual value of the output is $y_{i}$. The error between the actual value (target) and the model output is $\varepsilon$. The error is assumed to obey a normal distribution, i.e.; $\varepsilon_{i} \sim N(0,\;\sigma^{2} )$.

7 $$y_{i} = f(x_{i} ;\theta *) + \varepsilon_{i}$$

The least-squares estimation of the model output is $\hat{y}_{i}$

8 $$\hat{y}_{i} = f(x_{i} ;\hat{\theta })$$

The first-order Taylor expansion is conducted on $\hat{y}_{i}$ to obtain

9 $$\hat{y}_{i} = f(x_{i} ;\theta^{*} ) + f_{0}^{{\text{T}}} (\theta - \theta^{*} )$$

Therein, $f_{0}^{{\text{T}}}$ represents the Jacobian matrix

10 $$f_{0}^{{\text{T}}} = \left[ {\frac{{\partial f(x_{i} ;\theta^{*} )}}{{\partial \theta_{1}^{*} }},\frac{{\partial f(x_{i} ;\theta^{*} )}}{{\partial \theta_{2}^{*} }},...,\frac{{\partial f(x_{i} ;\theta^{*} )}}{{\partial \theta_{p}^{*} }}} \right]$$

The unbiased estimation of the variance of the error $\varepsilon$ is $s$, and it is expressed as follows:

11 $$s = \frac{1}{n - p}\sqrt {\sum\limits_{i = 1}^{n} {y_{i} - f(x_{i} ;\hat{\theta })} }$$

Student’s t-distribution is utilized. Then, the upper and lower bounds of the prediction intervals $U_{i}$ and $L_{i}$ are acquired. The notation $t_{n - p}^{1 - \alpha /2}$ indicates the quantile under the confidence level of $1 - \alpha /2$.

12 $$U_{i} = \hat{y}_{i} + t_{n - p}^{1 - \alpha /2} s\sqrt {1 + f_{0}^{T} (F^{T} F)^{ - 1} f_{0} }$$

13 $$L_{i} = \hat{y}_{i} - t_{n - p}^{1 - \alpha /2} s\sqrt {1 + f_{0}^{T} (F^{T} F)^{ - 1} f_{0} }$$

where $F$ denotes the Jacobian matrix

The PI coverage probability (PICP) and mean prediction interval width (MPIW) indices are introduced to describe the prediction intervals.

14 $$F = \left[ {\begin{array}{*{20}c} {\frac{{\partial f(x_{1} ,\;\hat{\theta })}}{{\partial \theta_{1} }}} & {\frac{{\partial f(x_{1} ,\;\hat{\theta })}}{{\partial \theta_{2} }}} & \cdots & {\frac{{\partial f(x_{1} ,\;\hat{\theta })}}{{\partial \theta_{p} }}} \\ {\frac{{\partial f(x_{2} ,\;\hat{\theta })}}{{\partial \theta_{1} }}} & {\frac{{\partial f(x_{2} ,\;\hat{\theta })}}{{\partial \theta_{2} }}} & \cdots & {\frac{{\partial f(x_{2} ,\;\hat{\theta })}}{{\partial \theta_{p} }}} \\ \vdots & \vdots & \cdots & \vdots \\ {\frac{{\partial f(x_{n} ,\;\hat{\theta })}}{{\partial \theta_{1} }}} & {\frac{{\partial f(x_{n} ,\;\hat{\theta })}}{{\partial \theta_{2} }}} & \cdots & {\frac{{\partial f(x_{n} ,\;\hat{\theta })}}{{\partial \theta_{p} }}} \\ \end{array} } \right]$$

15 $${\text{PICP}} = \frac{1}{n}\sum\limits_{i = 1}^{n} {c_{i} }$$

16 $${\text{MPIW}} = \frac{1}{n}\sum\limits_{i = 1}^{n} {(U_{i} - L_{i} )}$$

where *n* is the number of samples. ${\text{c}}_{i}$ is 1 when the prediction value falls within the bounds of the prediction intervals. Otherwise, it equals 0.

The normalized MPIW (NMPIW) is applied to indicate the width of the PIs. It is expressed as follows:

17 $${\text{NMPIW}} = \frac{{{\text{MPIW}}}}{L}$$

18 $$L = y_{\max } - y_{\min }$$

where $L$ is the difference between the maximum and minimum values of the target.

To determine the quality of the PIs, the PICP and NMPIW values need to be balanced. The ideal state is that the coverage probability of a PI is higher under a narrower interval width. Therefore, a combinational coverage criterion (CWC) is considered for comprehensive evaluation.

19 $${\text{CWC}} = {\text{NMPIW}}(1 + \gamma ({\text{PICP}})e^{{ - \eta ({\text{PICP}} - \mu )}} )$$

where $\mu$ denotes the confidence level and is usually equal to $1 - \alpha /2$. Moreover, $\gamma ({\text{PICP}})$ satisfies the following relationship.

20 $$\gamma = \left\{ \begin{gathered} 0,{\text{ PICP}} \ge \mu \, \hfill \\ 1,{\text{ PICP}} < \mu \hfill \\ \end{gathered} \right.$$

$\gamma ({\text{PICP}})$ is 0 when the PICP value exceeds the confidence level $\mu$, and then the CWC value is exactly the same as the ${\text{NMPIW}}$ value. When the PICP value is less than $\mu$, the NMPIW value need to be corrected by setting $\gamma ({\text{PICP}})$ to 1. $\eta$ is a penalty parameter, and a larger magnitude can better reflect the gap between PICP and $\mu$. $\eta$ is set to 50 in this paper. From Eq. (20), we can conclude that a small-magnitude CWC indicates high-quality PIs with low NMPIW and high PICP values.

### Real-time correction with the Kalman filter

#### Linear Kalman filter

**Figure 2:**
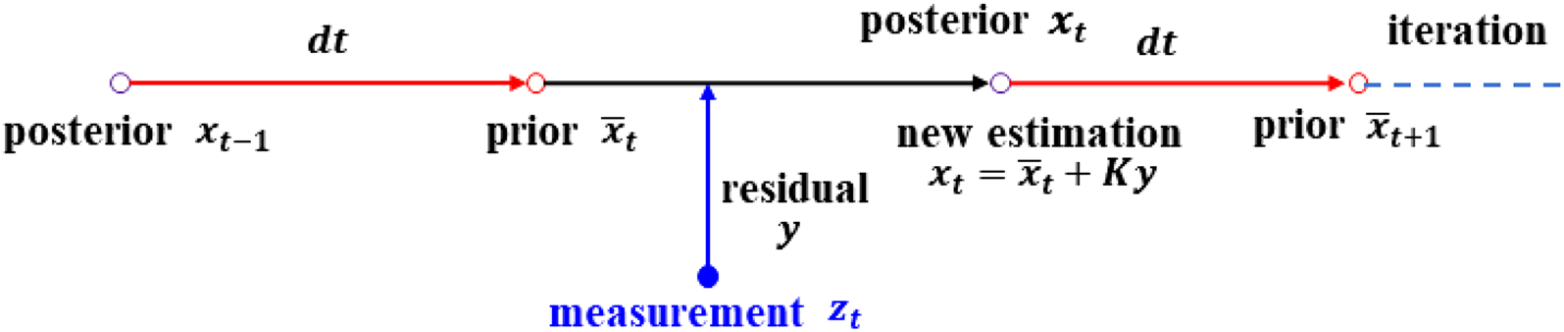
Diagram of the Kalman filter.

The KF method is mainly composed of prediction and update parts^59^. As shown in Fig. 2, the state $x_{t - 1}$ at moment *t*-1 is regarded as posteriori information. The prior state $\overline{x}_{t}$ is acquired after a prediction made on the basis of $x_{t - 1}$. Then, the measurement $z$ is fused in the estimation for updating. The residual between the measurement and priori state is assigned a weight *K*, which is named the Kalman gain. Then, the weighted residual is added to $\overline{x}_{t}$, and it becomes the new posteriori at moment* t* + 1. In this case, the prediction value is corrected by measurement in real time. The main formula of the KF is as follows.

The prediction process is as follows:

21 $$\overline{x} = Fx + Bu$$

22 $$\overline{P} = FPF^{T} + Q$$

The Eq. (21) is named the process model or state model. $x$ and $P$ indicate the state mean and covariance. $F$ is the state transition matrix, and $Q$ is the process noise. $B,u$ are input control parameters.

The update process is as follows:

23 $$y = z - H\overline{x}$$

24 $$K = \overline{P}H^{T} (H\overline{P}H^{T} + R)^{ - 1}$$

25 $$x = \overline{x} + Ky$$

26 $$P = (I - KH)\overline{P}$$

where $H$ is the measurement matrix and transforms the state to a measurement. $z$ is the measurement mean, and $R$ is the noise covariance. $y$ is the residual, and K denotes the Kalman gain.

#### Unscented Kalman filter

The unscented Kalman filter (UKF) is applied when the process model (Eq. 21) or the measurement model (Eq. 23) is nonlinear. This method makes no assumptions about the data distribution. Sigma points are generated and assigned weights to approach the real state. Then, they are transformed after the unscented transform. The mean and covariance of the transformed points are calculated as a new estimation of the state. Similar to the KF, the UKF contains two components of prediction and updating. The main steps are as follows:

(1) Generate sigma points.

The sigma points are symmetrically distributed. The central point $\mathcal{X}_{0}$ is assigned as the mean of input $\mu$. The remaining points are situated on both sides, and they satisfy^60^.

27 $$\chi_{i} = \left\{ \begin{gathered} \mu + [\sqrt {(n + \lambda )\Sigma } ]_{i} ,\;i = 1,\;...,\;n \hfill \\ \mu - [\sqrt {(n + \lambda )\Sigma } ]_{i - n} ,\;i = (n + 1),\;...,\;2n \hfill \\ \end{gathered} \right.$$

where $\lambda$ equals $\alpha^{2} (n + \kappa ) - n$ and $\alpha$ and $\kappa$ are constants. $n$ is the dimension of the state vector.

The weights of the mean and covariance of the central sigma point are $w_{0}^{m}$ and $w_{0}^{c}$, respectively.

28 $$w_{0}^{m} = \frac{\lambda }{n + \lambda }$$

29 $$w_{0}^{c} = \frac{\lambda }{n + \lambda } + 1 - \alpha^{2} + \beta$$

The weights of the points on the two sides are $w_{i}^{m}$ and $w_{i}^{c}$. They are computed with the following equation, where $i$ is the sequence number of (2*n* + 1) sigma points (except for the central point) in an *n*-dimensional problem.

30 $$w_{i}^{m} = w_{i}^{c} = \frac{1}{2(n + \lambda )},\;i = 1,\;...,\;2n$$

(2) The prediction step.

The process model is shown in Eq. (27). Then, the sigma points become transformed points, named $\mathcal{Y}$.

31 $$\mathcal{Y} = f(\mathcal{X})$$

The mean $\overline{x}$ and covariance $\overline{P}$ of the transformed points are calculated in Eqs. (31) and (32). This process is named the unscented transform^61^. The information acquired after the unscented transform is a priori.

32 $$\overline{x} = \sum\limits_{i = 0}^{2n} {w_{i}^{m} } \mathcal{Y}_{i}$$

33 $$\overline{P} = \sum\limits_{i = 0}^{2n} {w_{i}^{c} (} \mathcal{Y}_{i} - \overline{x})(\mathcal{Y}_{i} - \overline{x})^{{\text{T}}} + Q$$

(3) Update step.

The measurement model is as follows^62^.

34 $$\mathcal{Z} = h(\mathcal{Y})$$

The mean $\mu_{z}$ and covariance $P_{z}$ of the measurement are also calculated with an unscented transform.

35 $$\mu_{z} = \sum\limits_{i = 0}^{2n} {w_{i}^{m} } \mathcal{Z}_{i}$$

36 $$P_{z} = \sum\limits_{i = 0}^{2n} {w_{i}^{c} } (\mathcal{Z}_{i} - \mu_{z} )(\mathcal{Z}_{i} - \mu_{z} )^{{\text{T}}} + R$$

The residual $y$ between the measurement $z$ and prediction $\mu_{z}$ can be determined as follows:

37 $$y = z - \mu_{z}$$

The cross-covariance of the measurement and state is denoted as $P_{xz}$.

38 $$P_{xz} = \sum\limits_{i = 0}^{2n} {w_{i}^{c} } (\mathcal{Y}_{i} - \overline{x})(\mathcal{Z}_{i} - \mu_{z} )^{{\text{T}}}$$

Then, we compute the Kalman gain $K$ and obtain the updated covariance $P$.

39 $$K = P_{xz} P_{z}^{ - 1}$$

40 $$P = \overline{P} - KP_{z} K^{T}$$

The state is updated in Formula (41), and the posteriori information is acquired.

41 $$x = \overline{x} + Ky$$

Generally, the UKF method generates sigma points to avoid making assumptions about the data distribution. Thus, the UKF can be applied to nonlinear systems. The UKF method uses deterministic sampling to capture the statistical characteristics (the mean and covariance) of systems, and it is more adaptive to highly nonlinear problems^39^.

In this paper, the aforementioned method is employed to predict the settlement of the upper dam at the Qingyuan pumped-storage power station in Guangdong Province, China. The measurement data of the embankment settlement are first characterized by trend identification with wavelet analysis. It helps to determine the major factor influencing embankment settlement. Then, the Delta method is applied to generate PIs based on the new database. Then, the KF and UKF are implemented to correct the prediction output and the bounds of the PIs. It is regarded as the development of real-time correction from point forecasting to prediction intervals.

## Settlement prediction for the dam at the Qingyuan power station

**Figure 3:**
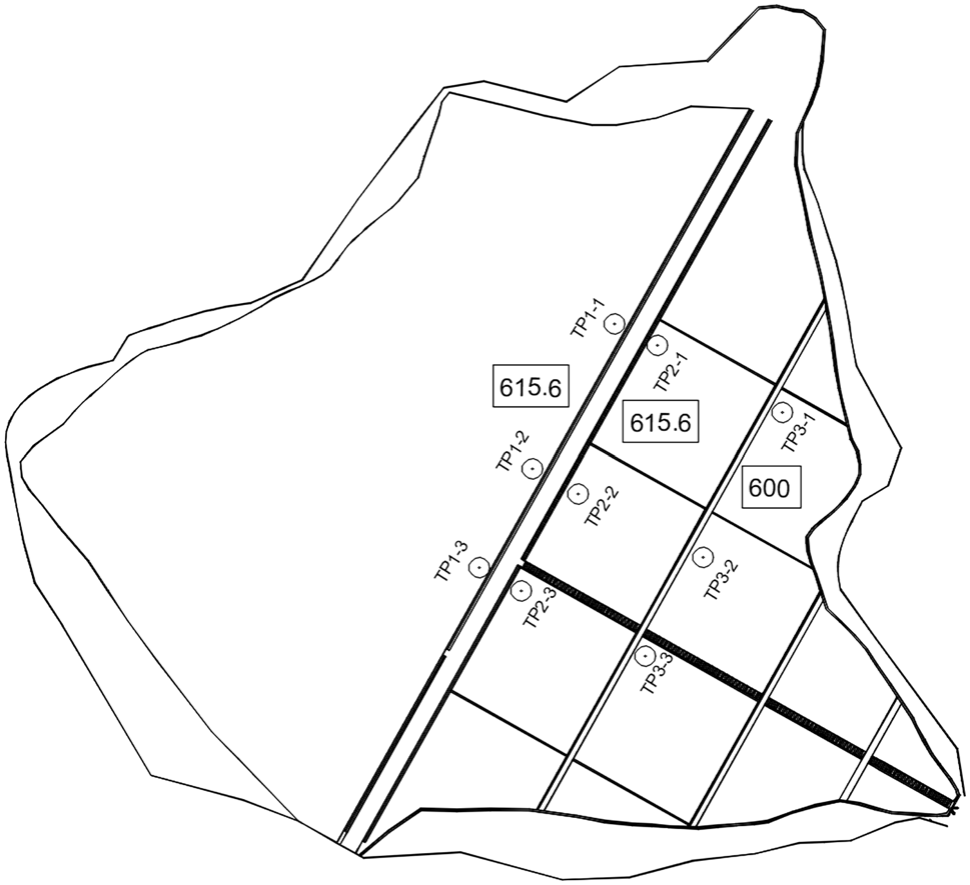
Layout of the controlled points (from TP1-1 to TP3-3).

The main and auxiliary dams of the Qingyuan pumped-storage power station in Guangdong Province are rockfill dams with clay core walls. The height of the dam crest is 615.6 m. The upper reservoir began to be filled in February 2011 and was used for storage in April 2013. The lower reservoir was used for storage in August 2014. All 4 units were put into production in August 2016. We analyze the crest settlements of the Qingyuan dam with nine control points located on the upper reservoir (points TP1-1 to TP 3–3, see Fig. 3). Each point returns 51 settlement data samples from April 7, 2013, to February 1, 2019.

### Trend identification and preprocessing

The settlement of the dam is affected by many factors. The factors can be described as creep $\delta_{\theta }$, construction $\delta_{d}$, water pressure $\delta_{h}$, temperature $\delta_{T}$, and noise $\delta_{\varepsilon }$. Thus, the general settlement $\delta$ can be expressed with the following function, which is usually nonlinear.

42 $$\delta { = }f(\delta_{d} ,\delta_{h} ,\delta_{\theta } ,\delta_{T} ,\delta_{\varepsilon } )$$

**Figure 4:**
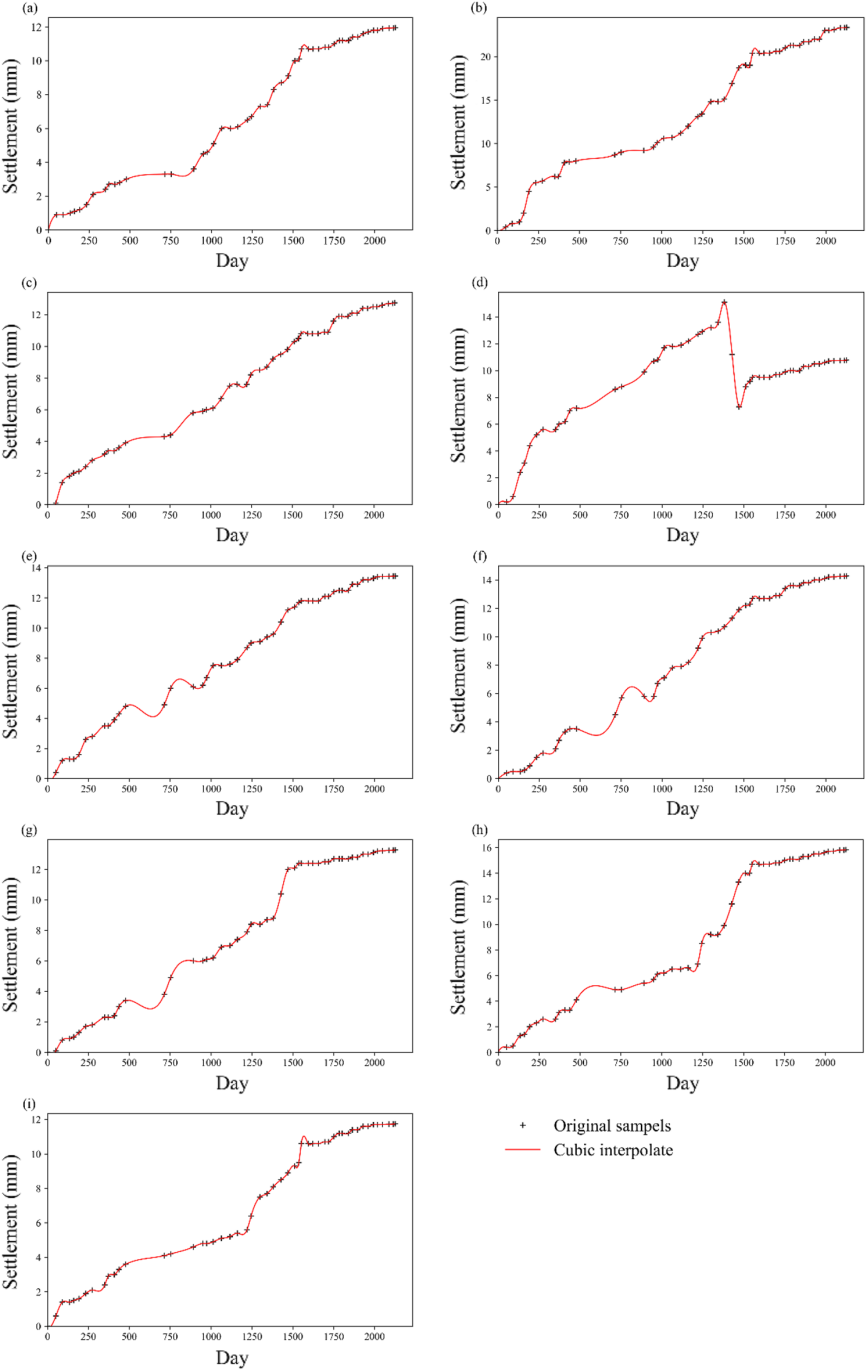
Cubic spline interpolation results of the settlement data: (**a**) TP1-1; (**b**) TP1-2; (**c**) TP1-3; (**d**) TP2-1; (**e**) TP2-2; (**f**) TP2-3; (**g**) TP3-1; (**h**) TP3-2; and (**i**) TP3-3.

To address the time discontinuity, cubic spline interpolation was applied, and the interval was one day. The interpolated settlement curve is smooth (see Fig. 4), and we acquire 2125 data samples at each point. SOM clustering and mean change point analysis are conducted, and the construction period is analyzed. Finally, the data after 1220 days are considered for future modeling to remove the unstable settlement information during the filling period. Therefore, the effect of the construction components $\delta_{d}$ on the settlement after 1220 days is negligible. It is noted that the period of the monitored data is almost 5 years, and the settlement was not affected by specific temperatures. Thus, the component $\delta_{T}$ does not influence the prediction.

**Figure 5:**
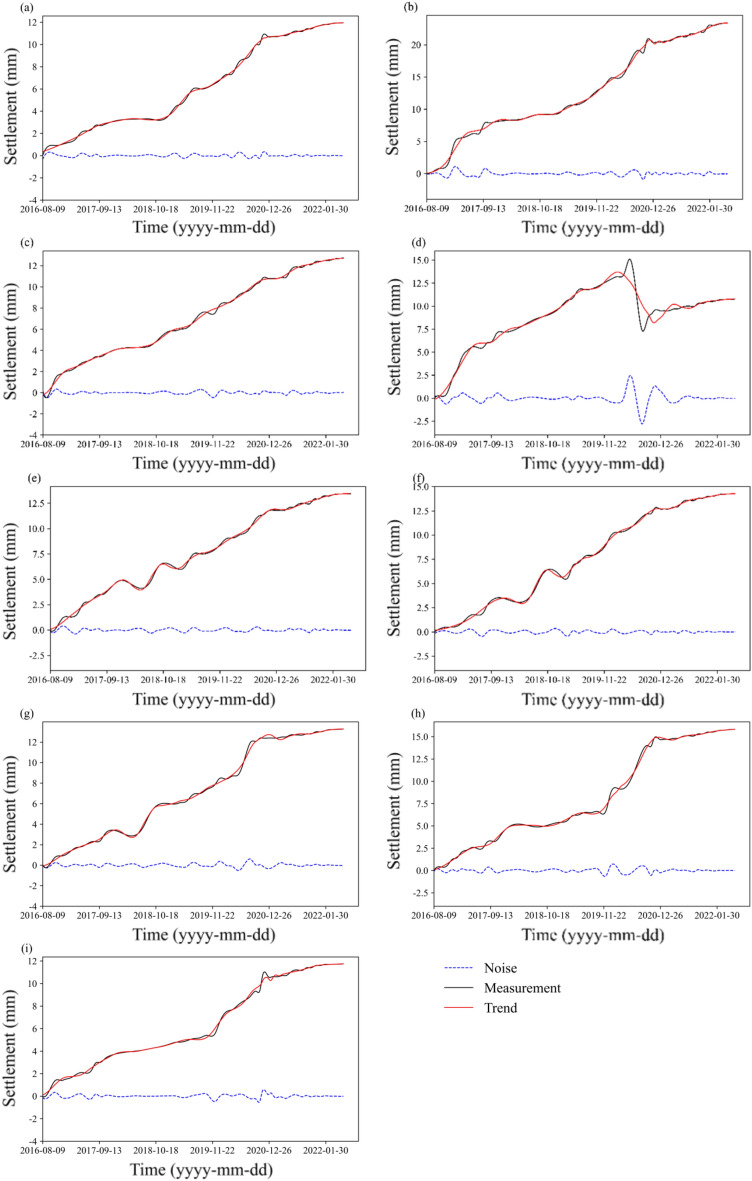
Settlement data processing results of the model with wavelet analysis: (**a**) TP1-1; (**b**) TP1-2; (**c**) TP1-3; (**d**) TP2-1; (**e**) TP2-2; (**f**) TP2-3; (**g**) TP3-1; (**h**) TP3-2; and (**i**) TP3-3.

The original settlement data of the nine points are discontinuous in time with multiple tips and fluctuations (see the black marks in Fig. 4). Hence, trend identification and preprocessing are needed for further calculation. The Daubechies (Db7) wavelet is chosen to obtain the settlement trend, and the results are shown in Fig. 5. The original measurement data are decomposed at the maximum level. The trend is acquired by reconstructing the decomposed components, eliminating noise with high frequency. The red trend curve in Fig. 5 becomes smoother than the original measurement data. It can be seen from the figure that the local fluctuations are filtered without altering the general trend. In this regard, the noise data are separated, and the effect of the noise components $\delta_{\varepsilon }$ can be removed. The local fluctuations caused by water pressure $\delta_{h}$ are also eliminated.

Based on the description above, we obtain the tendency of the settlement without noise, fluctuations, unstable displacements, etc. The major influencing factor of the settlement is the creep component $\delta_{\theta }$. Then, we can take the constitutive relationship into consideration for prediction rather than merely data processing. In this study, the powder-type creep equation is utilized according to the settlement trend. The equation is suitable for the nonlinear viscoelastic problem^63,64^.

43 $$\dot{\varepsilon } = A\sigma^{n} t^{m}$$

where $\dot{\varepsilon }$ indicates the creep strain rate and $\sigma$ (MPa) is the equivalent stress. $t$ (days) is the creep time. $A, \, m, \, n$ are the fitted constants. We apply the integral form in the later calculation.

44 $$\varepsilon = c + \frac{{A\sigma^{n} t^{m + 1} }}{m + 1}$$

where $c$ is an integral constant.

### PI construction

**Figure 6:**
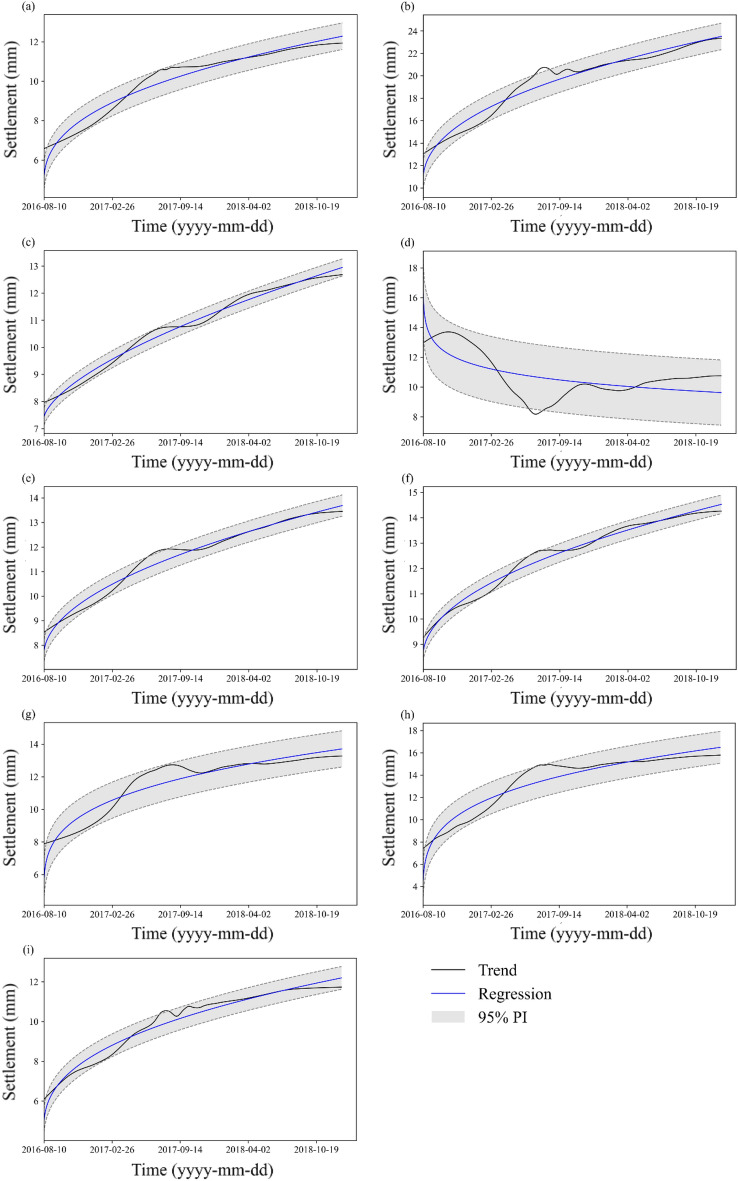
95% prediction interval of the regression model: (**a**) TP1-1; (**b**) TP1-2; (**c**) TP1-3; (**d**) TP2-1; (**e**) TP2-2; (**f**) TP2-3; (**g**) TP3-1; (**h**) TP3-2; and (**i**) TP3-3.

According to the creep model in Eq. (44), the Delta method is applied to calculate the 95% prediction interval of each control point (see Fig. 6). Most of the measurement data are situated in PIs. Moreover, the upper and lower bounds of the PIs are smooth curves that do not fluctuate with the measurement.

The PI parameters of each point are listed in Table 1. The results show that the NMPIW value of the PIs is below 0.316, and the PICP value is over 90%. The comprehensive index CWC value is generally low. This indicates that the constructed PIs are of high quality with narrow widths and high coverage. The maximum CWC value appears at TP2-1 among all points due to the abnormal increase in the monitoring data. It is noted that the value of NMPIW is the same as the CWC value at some points since their PICP values reach the confidence level (95%), and then no penalty is carried out.

**Table 1:** Evaluation indices of the PIs and creep model parameters at the control points.

Points	PICP	NMPIW	CWC	*A*	*m*	*n*	*c*
TP1-1	0.990	0.116	0.116	2.629	− 0.601	21.313	4.704
TP1-2	0.943	0.101	0.246	2e − 4	− 0.558	5.673	10.441
TP1-3	0.990	0.049	0.049	0.084	− 0.364	18.146	7.373
TP2-1	0.949	0.316	0.656	− 0.004	− 0.966	8.842	41.934
TP2-2	0.929	0.065	0.249	0.033	− 0.492	15.525	7.604
TP2-3	0.954	0.052	0.052	0.039	− 0.511	15.666	8.492
TP3-1	0.992	0.167	0.167	0.008	− 0.721	11.131	4.356
TP3-2	0.966	0.182	0.182	0.075	− 0.746	13.799	1.938
TP3-3	0.963	0.098	0.098	6e − 4	− 0.607	8.094	4.478

**Figure 7:**
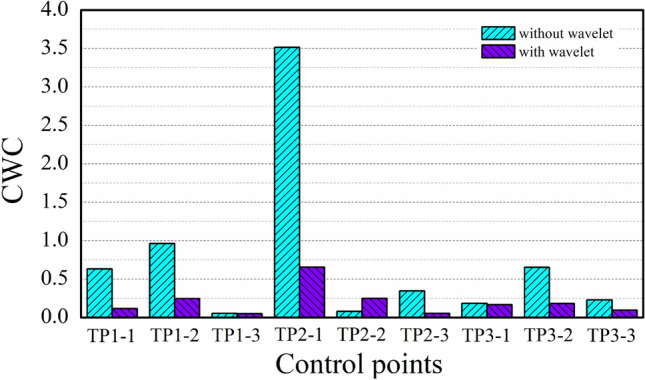
Comparison of the CWC values of PIs before and after wavelet analysis.

A comparison of the CWC index of PIs with and without wavelet analysis is illustrated in Fig. 7. The results show that the CWC values at eight points decrease after wavelet analysis, especially at point 2–1. According to the above description, the CWC value reflects the general value of the coverage and width of PIs, and a lower value indicates higher prediction quality. It reveals that the PIs constructed after trend identification using wavelet analysis are more accurate than those constructed based on the original data.

**Figure 8:**
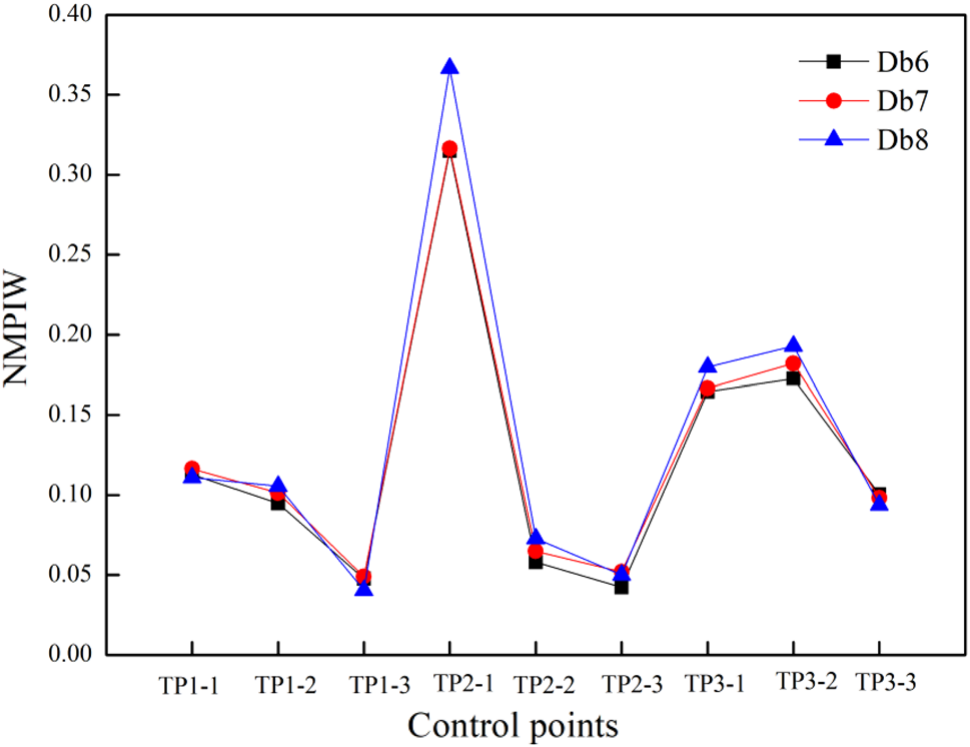
NMPIW value of PIs with Db6, Db7 and Db8 wavelets at different control points.

Db1-10 wavelets are commonly used in signal analysis and processing, and a larger number indicates a longer and smoother wavelet. In this case, the NMPIW value of the PIs based on different lengths of Db wavelets (i.e., Db6-8) are compared in Fig. 8. The NMPIW value increases with increasing wavelet length. The phenomenon reveals that longer wavelets can retain more details (noise data), and then the width of a PI becomes larger to include the extra information completely. However, wavelets that are too short ignore more details and build a relatively narrow PI that can hardly cover the settlement trend. Therefore, Db7 wavelets are recommended for constructing PIs of embankment settlement data.

**Figure 9:**
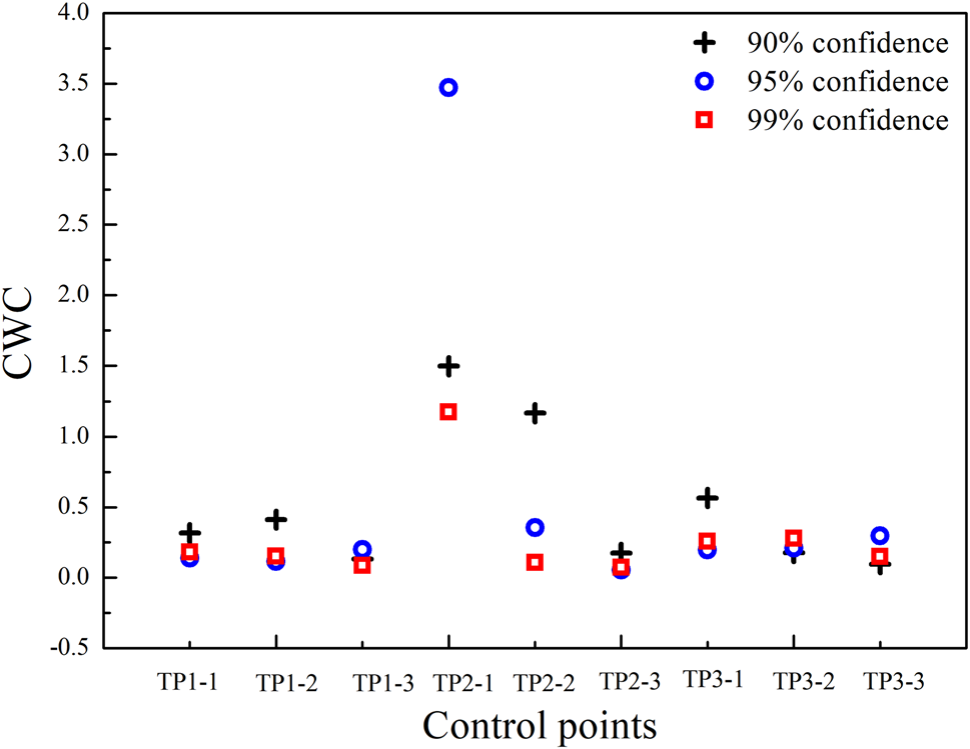
CWC values of PIs at different confidence levels.

The CWC values of the PIs with confidence levels of 90%, 95%, and 99% are calculated. The results are plotted in Fig. 9. The CWC values of the 90% prediction interval are higher than those of the 95% and 99% intervals at 5 points. However, there was no consistent rule at all control points. This phenomenon reveals that the prediction evaluation may not merely depend on higher confidence levels. The 95% confidence level can meet the needs in most conditions.

### Real-time correction prediction result

Then, the UKF method is applied to correct the value of the regression output by incorporating the actual measurement from each day. The state vector of the system takes $\varepsilon_{k}$ (i.e., the strain at time $t_{k}$) with the expression in Eq. (45) according to the time hardening creep model. Then, the iteration relationship between strain $\varepsilon_{k + 1}$ at time $t_{k + 1}$ and $\varepsilon_{k}$ can be calculated in Eq. (47), which is the nonlinear process model of the UKF.

45 $$\varepsilon_{k} = c + \frac{{A\sigma^{n} t_{k}^{m + 1} }}{m + 1}$$

46 $$t_{{k{ + }1}} { = }t_{k} + dt$$

47 $$\varepsilon_{k + 1} = \frac{{A\sigma^{n} }}{m + 1}(\sqrt[{(m + 1)}]{{\frac{{(\varepsilon_{k} - c)(m + 1)}}{{A\sigma^{n} }}}} + dt)^{m + 1} + c$$

The measurement model needs a transformation from displacement to strain (Eq. 48) since the measured data in this case are displacement data, where $\overline{H}$ is the equivalent height of the dam and $y$ is the displacement vector.

48 $$y = \overline{H} \varepsilon$$

The initial state is set as the primary regression output (e.g., 12.29 at point TP1-1). The initial P value is set to 1. The process covariance Q is 1, and the measurement covariance R is 10. The KF and EKF methods are also complemented with the same Q and R values for a fair comparison with the UKF. The process model of the KF is expressed is linear form in Eq. (49), where $$\overset{\lower0.5em\hbox{$\smash{\scriptscriptstyle\frown}$}}{\varepsilon }_{k}$$ is the output vector of the regression model.

49 $$\varepsilon_{k + 1} = \overset{\lower0.5em\hbox{$\smash{\scriptscriptstyle\frown}$}}{\varepsilon }_{k}$$

The prediction results are evaluated by comparing the prediction intervals with the trend settlement data over the last 30 days. As shown in Fig. 9, most trend data fall into the 95% PIs. The general prediction effect is good, and the prediction ratio can reach 100% at 8 points and 53% at TP1-3. The prediction effect is worse at TP1-3 than at the other points. To determine the reason, we found that the fluctuations in the trend data of TP1-3 have small amplitudes and large wavelengths, and they are unsuitable to be regarded as noise (high frequency). Consequently, the width of the PI constructed at such a trend is the narrowest among all the points, which may decrease the prediction coverage. For the real-time correction effect, it is found that the KF-modified curve is situated between the original regression curve and trend curve. The UKF-modified curve rapidly converges to the measurement curve. Generally, the modified prediction output is closer to the trend data during prediction, and the performance of the UKF is better than that of the KF.

**Figure 10:**
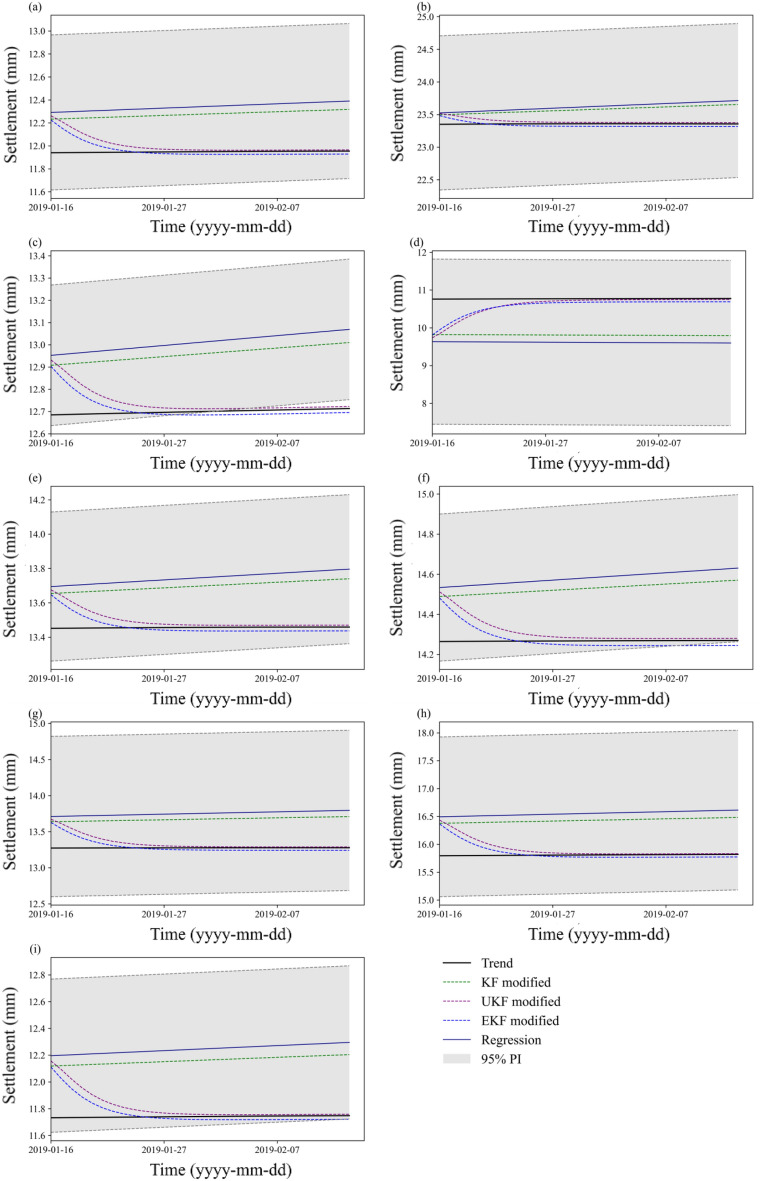
Prediction effect of PIs and modified outputs by KF, EKF, and UKF: (**a**) TP1-1; (**b**) TP1-2; (**c**) TP1-3; (**d**) TP2-1; (**e**) TP2-2; (**f**) TP2-3. (**g**) TP3-1; (**h**) TP3-2; and (**i**) TP3-3.

**Figure 11:**
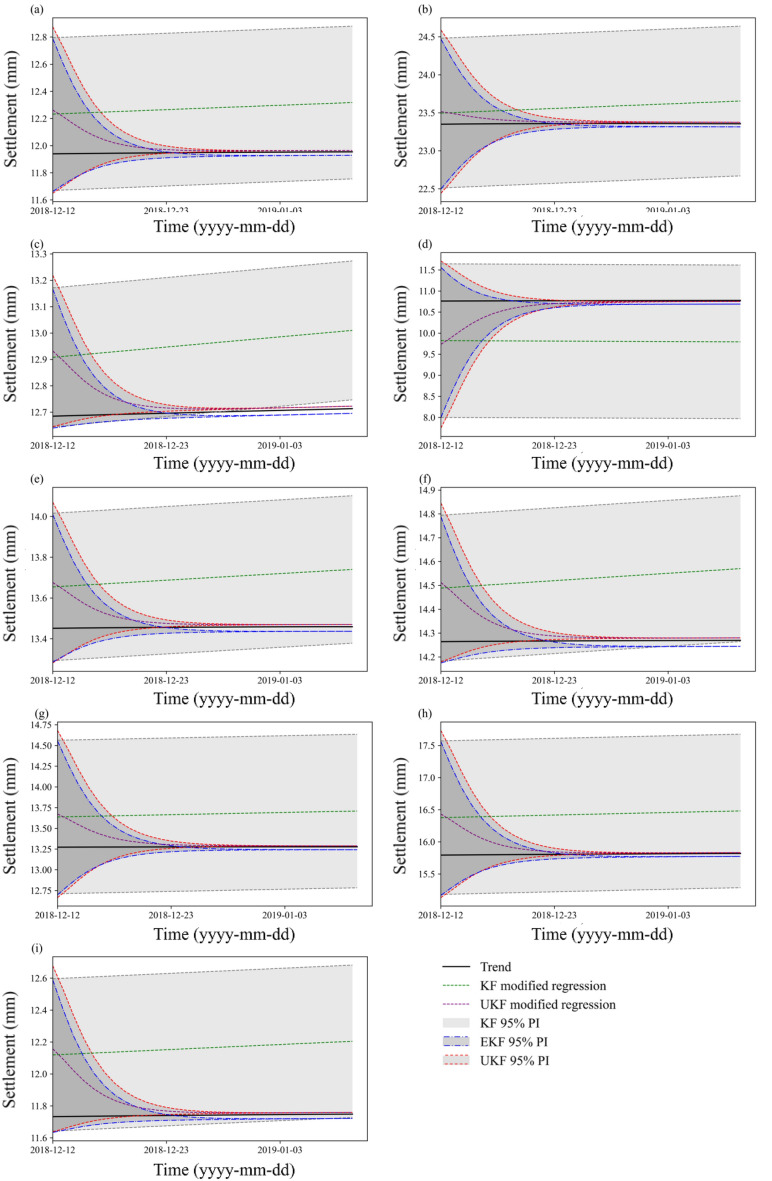
PIs with real-time correction by KF, UKF and EKF: (**a**) TP1-1; (**b**) TP1-2; (**c**) TP1-3; (**d**) TP2-1; (**e**) TP2-2; (**f**) TP2-3; (**g**) TP3-1; (**h**) TP3-2; and (**i**) TP3-3.

In this paper, we also apply a straightforward approach to estimate the bounds of PIs in real time. The traditional time-invariant PIs are first constructed on historical data and shown in Fig. 10, where the UKF-modified regression curve is found to be closer to the actual measurements than the curves produced by KF. The root mean square error (RMSE) of the UKF, KF and regression output is also listed in Table 2. This indicates that the UKF has the smallest deviation from the actual measurement data. Then, we update the upper and lower bounds of the PIs in Fig. 10 by incorporating new information iteratively; thus, we obtain the real-time updated PIs in Fig. 11. The 95% PIs are updated and modified by the UKF and compared with those modified by the KF and EKF methods, as shown in Fig. 11. It is noted that the first values of the curves in Figs. 10, 11 indicate the updated state (not the initial state) after one-step calculation, and they show the differences due to the different filter accuracies. For a better comparison, the initial state and covariance matrix of different filters are set to the same value. The results show that the depth of the UKF-corrected PIs is significantly narrower than that of the KF-modified PIs without a loss of prediction precision compared with the time-invariant PIs. The UKF-modified PIs show similar convergence performance to the EKF-modified PIs in the earlier stage , while their deviation from the measurement trend is less than that of the EKF (especially in Fig. 11(c), (e), (f), (i)) as time proceeds. The results indicate that the UKF method can decrease the previously estimated model uncertainties and provide accurate PIs in real time. It is adaptive to the nonlinear system of embankment settlement prediction.

**Table 2:** The RMSE of the primary regression output (RMSE1), KF-modified output (RMSE2) and UKF-modified output (RMSE3).

Points	RMSE1	RMSE2	RMSE3
TP1-1	2.154	1.795	0.547
TP1-2	1.472	1.226	0.307
TP1-3	1.713	1.427	0.423
TP2-1	6.324	5.270	1.722
TP2-2	1.592	1.327	0.387
TP2-3	1.730	1.442	0.426
TP3-1	2.615	2.179	0.676
TP3-2	4.087	3.406	1.073
TP3-3	2.768	2.307	0.716

## Conclusions

This paper proposes a real-time corrected prediction interval method for embankment settlement monitoring. The method can construct time-varying PIs by the UKF algorithm to decrease prediction uncertainties iteratively without the need for reconstruction models. Primarily, trend identification using wavelet analysis is proposed to determine the major factor that influences the long-term trend of embankment settlement. Then, the Delta method is applied to construct PIs based on the creep equation established on the characterized trend. In this case, the prediction uncertainties can be estimated. The time-varying PIs are updated by the UKF using the new data to narrow the deviation between the prediction and measurement. The linear KF and EKF are also implemented as a comparison. The general conclusions are as follows:

(1) The data after wavelet analysis can reveal the general tendency of embankment settlement after eliminating unstable settlement information, local fluctuations, and the noise of the original data. Then, 95% PIs are constructed by the Delta method with high coverage and small widths. The envelope of the PIs is smoother and has less fluctuation than the original measurement. The PIs reveal the general trend of dam settlement and can be used for effective prediction.

(2) Thirty days of measurement data are used to evaluate the prediction effect of the PIs. The results show that the measurement is generally included in the PIs at nine control points. It is worth noting that the prediction ratio remains at 100% for the eight points even with abnormal local uplift (TP2-1). The prediction ratio is 53% at TP1-3, which reflects the balance of the PI width and prediction coverage. The result indicates that the PIs generated in this study contain the uncertainty estimation of prediction and are not affected by local anomaly data.

(3) The actual measurement is applied to correct the prediction output and the envelope of the PIs by the UKF method, and the effect is compared with the effects of the KF and EKF. The UKF-corrected output is closer to the measurement, and the modified PIs are narrower with high coverage. The results show that the real-corrected prediction interval by the UKF is more consistent with the actual results. The approach that combines both the prediction model and measurement data mining, which are promising for accurate prediction.

Recently, the development of machine learning and artificial intelligence methods has changed many fields, including geotechnical engineering. Intelligent methods, such as NNs and ELMs, have become promising techniques in PI estimation due to their strong learning abilities. It is thought that further work will focus on the feed-forward updating techniques of intelligent methods for real-time covariance estimation. In addition, due to the complexity and nonlinearity of the practical embankment settlement problem, the improved UKF (such as the adaptive scheme) methods need to be further investigated and their performance and feasibility need to be compared with other updating algorithms.

## Data Availability

The datasets used and/or analysed during the current study available from the corresponding author on reasonable request.
